# BIOMARKERS HELP US UNDERSTAND HOW CELLULAR AND SYSTEMIC AGING CONTRIBUTE TO MORTALITY

**DOI:** 10.1093/geroni/igae098.1220

**Published:** 2024-12-31

**Authors:** Eric Klopack, Eileen Crimmins

**Affiliations:** University of Southern California, University of Southern California, California, United States; University of Southern California, Los Angeles, California, United States

## Abstract

Research suggests aging is a coordinated decline occurring in multiple systems and at multiple biological levels. However, it is largely unknown how general biological aging and specific systemic aging co-occur and influence one another to affect health outcomes like mortality, multimorbidity, and functional limitations. There is also emerging interest in understanding how social exposures may differentially accelerate decline in individual physiological systems. We utilize data from the Health and Retirement Study, a nationally representative sample of about 4000 US adults over age 55. We used eXtreme Gradient Boosting (xgboost) in a training subsample to create estimates of health risk based on mortality, multimorbidity, and functional limitations using sets of biomarkers representing biological systems (e.g., nervous, immune, cardiovascular, and renal systems) as well as cellular aging (e.g., epigenetic clocks). These models generated risk scores for each outcome based on each system (e.g., renal functioning using BUN, creatinine, and cystatin C). Outcomes were regressed on these scores in a testing subsample to assess the relative impact of each system on health. Results suggest cellular aging, respiratory aging, and metabolic dysfunction contribute most to these health outcomes. Aging of these systems also partially mediates the risk associated with age, race/ethnicity, sex, and education. These results suggest aging of specific systems may be differentially affected by sociodemographic exposures and may differentially contribute to health risks. This research may help guide interventions focused on reducing inequalities in mortality risk by identifying health risks that would have the greatest impact on health at the population level.

